# Self-rated Health in Youth with Different Screen Time in Their Adolescence: Tehran Lipid and Glucose Study

**DOI:** 10.34172/aim.2023.99

**Published:** 2023-12-01

**Authors:** Parnian Parvin, Fatemeh Mahani, Leila Cheraghi, Reza Yari-Boroujeni, Fereidoun Azizi, Parisa Amiri

**Affiliations:** ^1^Research Center for Social Determinants of Health, Research Institute for Endocrine Sciences, Shahid Beheshti University of Medical Sciences, Tehran, Iran; ^2^Department of Epidemiology and Biostatistics, Research Institute for Endocrine Sciences, Shahid Beheshti University of Medical Sciences, Tehran, Iran; ^3^Endocrine Research Center, Research Institute for Endocrine Sciences, Shahid Beheshti University of Medical Sciences, Tehran, Iran

**Keywords:** Adolescents, HRQoL, Physical activity, Screen time, Sedentary life, Youth

## Abstract

**Background:**

The long-term effects of childhood screen time on health-related quality of life (HRQoL) are still unclear. This study aimed to investigate the relationship between screen time during adolescence and sex-specific HRQoL in early youth.

**Methods:**

We studied the data from 642 adolescents aged 13-19 years, who participated in the Tehran Lipid and Glucose Study from 2005 to 2011 (baseline) with complete data on HRQoL in their early adulthood (22-28 years at the last follow-up). Physical and Mental HRQoL were assessed using the Iranian version of the short-form 12-item health survey version 2 (SF-12v2). Screen time and leisure-time physical activity were evaluated using the Iranian Modifiable Activity Questionnaire (MAQ). All analyses were conducted in Stata (version 14); MI used the mi impute command.

**Results:**

The mean±SD of age, body mass index (BMI), and physical activity in childhood were 16.33±1.27, 23.27±4.63 and 13.77±16.07, respectively. Overall, 35% of boys and 34% of girls had high screen time (HST) in childhood. In general, the HRQoL scores in male participants were higher than in females in both the mental and physical domains. HST in males in childhood was associated with decreased mental health (β=-6.41, 95% CI: -11.52, -1.3 and *P*=0.014), social functioning (β=-5.9, 95% CI: -11.23, -0.57 and *P*=0.03) and mental component summary (MCS) (β=-2.86, 95% CI: -5.26, -0.45 and *P*=0.02). The odds of poor MCS were significantly higher in those with HST compared to their counterparts with low screen time (LST) after adjusting for all potential cofounders.

**Conclusion:**

The results of the present study showed the negative effect of screen time during adolescence on HRQoL in early youth. This effect was observed in men, mainly in the mental dimension. Investigating the long-term consequences of screen-time behaviors on self-assessed health in other populations with the aim of effective primary prevention is also suggested.

## Introduction

Sedentary behaviors, as activities with low energy expenditure consisting of sitting, lying down, and screen-based entertainment have shown a rising trend during the last decades.^1-3^ In this regard, watching screens, including smartphones, tablets, gaming consoles, and televisions, occupies most of the children’s leisure time and is considered the most prevalent form of sedentary behavior in developed and developing countries.^4,5^ Like many countries, screen time in Iranian children and adolescents has also been significant due to the expansion of urbanization and its consequences such as living in apartments without yards and lack of safe green spaces with sports equipment.^6,7^ Based on a study conducted on nationally representative data, the screen time of 33.4% and 53% of Iranian students was more than 2 hours a day during school days and holidays, respectively.^8^ The available evidence has shown the negative impact of high screen time (HST) on physical and psychological health in childhood. In this regard, children and adolescents with high daily screen time are more likely to be obese, nervous, and irritable and have experienced poor health-related quality of life (HRQoL).^9-12^

HRQoL, as a multidimensional concept, reflects the individuals’ perception of their health status in physical, psychological, and social aspects.^13^ The negative effects of HST on various aspects of health from children’s perspective, which is measured in terms of HRQoL, are still controversial. While some studies have reported a significant adverse effect and association,^14,15^ fewer studies reject this relationship.^16,17^ According to a recent study in Iran, children and adolescents with more screen time and less physical activity reported poorer HRQoL than their peers with the opposite habits.^15^ Other studies also confirmed that adolescents who were physically active and had low screen time (LST) were more likely to report higher quality of life.^14-18^

Despite previous studies investigating the association between childhood HST and HRQoL in the short-term, none followed them into young adulthood and reported the effect. The effects of inactivity in the early years of life are not limited only to that period, and there is more evidence of a higher prevalence of physical and psychological problems in sedentary people throughout life.^19^ On the other hand, since early learning is likely to be preserved in the life course, childhood behavioral habits play an essential role in the formation of adulthood behaviors.^20,21^ As a result, childhood habits such as screen time can be used as a determinant and predictor for an adult’s sedentary lifestyle and its effect on their health.^22^ Hence, the present retrospective study, for the first time, investigated the relationship between screen time during adolescence and the individuals’ HRQoL in early youth in the context of the Tehran Lipid and Glucose Study (TLGS).

## Materials and Methods

###  Study Design and Participants 

The current study was conducted in the TLGS framework, an ongoing large-scale, family-based study performed in district 13 of Tehran, the capital of Iran. The mentioned region was chosen mainly because of the high stability of the region and also the representative age distribution of the entire population of Tehran. The rationale and design of the TLGS are previously published.^23^ Briefly, the TLGS consists of two main parts: a cross-sectional baseline assessment to examine the prevalence of non-communicable diseases (NCDs) and associated risk factors (1999-2001), and a subsequent follow-up study with lifestyle intervention. A multi-stage cluster random sampling method was used to select the participants. In the first stage, three centers were selected out of 20 healthcare centers in the 13^th^ district of Tehran. In the second stage, 7193 families were randomly invited to participate in the study. Among them, 4751 families (15 005 residents aged ≥ 3 years) agreed to participate in the TLGS, and along with them, 832 children aged 3 to 10 years were also included in the study. Participants were asked to attend a follow-up re-examination every three years and a total of five data re-collections were carried out between 2001 and 2018.^24,25^

 In the present analysis, 642 adolescents aged 13 to 19 years who participated in the third and fourth follow-up examinations of the TLGS, with complete data on their quality of life in early adulthood (the last follow-up examination) were evaluated. The mentioned participants were aged 3 to 10 years during baseline examination of the TLGS.

## Measurements

Trained interviewers utilized relevant questionnaires to collect socio-demographic data, including age, education, employment, and marital status. Education was categorized as primary, secondary, and higher. Employment status was divided into employed or unemployed. Marital status was considered as married and unmarried. Smoking was categorized as yes (current smokers) and no (past or never smokers). Weight status was defined as normal ( < 25 kg/m^2^), overweight (BMI ≥ 25 and BMI < 30), and obese (BMI ≥ 30). Leisure time physical activity (LTPA) and screen time (ST) were assessed using a reliable and validated Iranian version of the Modifiable Activity Questionnaire (MAQ).^26^ The questionnaire includes 15 popular and common Iranian activities specified for the mentioned age group during leisure time and time spent in each activity. As the main independent variable in the present study, screen time in adolescence was measured by asking “On average, how many hours do you spend each day watching TV (or videos) and playing video games (or computer)?”. Adolescents were divided into two groups: 1) mean ST < 2 hours/day, and 2) mean ST ≥ 2 hours/day as LST and HST, respectively.

Physical activity in adolescents was estimated based on related metabolic equivalent tasks (METs). Levels of physical activity were defined as low (MET < 600 min/wk), moderate (MET 600–2999 min/wk), and high (MET ≥ 3000 min/wk). MET is a physiological measure expressing the energy cost of physical activity, which is defined as the ratio of metabolic rate (or the rate of energy consumption) during a specific physical activity to a reference metabolic rate, set by convention to 3.5 mL O_2_ kg^-1^min^-1^.

HRQoL consists of two dimensions, physical and mental; data of each dimension was collected using the reliable and validated Iranian version of the short-form 12-item health survey version 2 (SF-12v2).^27^ The related physical and mental items are categorized into eight subscales: (1) Physical subscales including physical functioning, role limitations due to physical health problems, bodily pain, and general health; and (2) Mental subscales encompassing vitality, social functioning, role limitations due to emotional problems, and mental health. Subscale scores range from 0 (indicating the worst health condition) to 100 (which represents the best). Two summary scores, physical component summary (PCS) and mental component summary (MCS), are weighted representations of each domain.

###  Statistical Analysis

In the current study, the percentage of missing data ranged from 1% for BMI to 35% for LTPA values in childhood and from 1.4% for smoking to 38% for LTPA in adulthood. In terms of parental data, the range of missing data was from 7.2% to 19.2% for maternal age and paternal physical activity, respectively (Supplementary file 1, Table S1). To address the missing data, we used multiple imputations using chained equations (fully conditional specification).^28^ After the imputation of 10 datasets, the childhood and adulthood characteristics of individuals as well as their parental factors were compared between LST and HST groups for both sexes. The association between HRQoL in young men and women with different childhood screen time was assessed using linear regression analysis. In the current model, coefficient (β) represents the difference in the means of the HRQoL score for the HST group compared to the LST group. Unadjusted and adjusted logistic regression analyses were used to assess the association between screen time and poor HRQoL and the odds ratios (ORs) and 95% confidence intervals (CIs) were estimated for the HST group compared to the LST group, separately for men and women. We considered multiple imputations to estimate all confidence intervals and p-values. For each mental and physical domain, the poor HRQoL was defined as the first tertile of MCS or PCS, respectively. The second tertiles of the MCS and PCS scores were omitted from the analysis. Three models were considered as follows: Model 1 was unadjusted. Model 2 was adjusted only for the participant’s age, and model 3 was adjusted for the participant’s age, education, occupation, marital status, smoking, LTPA, and BMI. All analyses were carried out in Stata (version 14) (Stata Corp LP, College Station, TX USA); MI used the mi impute command.

## Results

In 642 participants (50.3% female), the mean ± SD values of age, BMI, and physical activity in childhood were 16.33 ± 1.27, 23.27 ± 4.63 and 13.77 ± 16.07, respectively. Moreover, 35% of boys and 34% of girls had HST in childhood. Baseline children and parental characteristics specified by gender and screen time status are presented in Table 1. For both sexes, there were no significant differences between ST groups regarding age, education, LTPA, and BMI (*P* > 0.005). In terms of parental characteristics, none of the factors that were examined in this study showed a significant difference between the ST groups in boys and girls.

**Table 1:** Adolescents’ and their Parents’ Characteristics According to Participants’ Sex and Screen Time: Tehran Lipid and Glucose Study, 2008-2013

	**Boys (n=323)**			**Girls (n=319)**		
	**LST**	**HST**	***P*** ** value**	**LST**	**HST**	***P*** ** value**
**Childhood**						
Age	16.31 ± 1.27	16.31 ± 1.25	0.987	16.25 ± 1.27	16.54 ± 1.26	0.073
Education (%)			0.432			0.537
Primary	63	58		58	55	
Secondary	37	42		42	45	
LTPA (Met/h/wk)	17.07 ± 15.61	16.79 ± 18.85	0.899	10.05 ± 12.91	10.87 ± 17.38	0.674
BMI	23.51 ± 4.74	23.01 ± 4.94	0.409	23.23 ± 4.35	23.15 ± 4.66	0.888
**Maternal Characteristics**						
Age	41.89 ± 5.28	42.93 ± 5.10	0.108	42.32 ± 5.16	42.67 ± 5.79	0.596
Education (%)			0.438			0.647
Primary	13	9		13	11	
Secondary	72	72		71	76	
Higher	15	19		16	13	
Job status (%)			0.600			0.931
Employed	17	14		15	14	
Unemployed	83	86		85	86	
Smoking (%)			0.946			0.503
Yes	4	4		2	1	
No	96	96		98	99	
Physical activity (%)			0.231			0.739
Low	38	42		19	18	
Moderate	12	18		25	22	
High	50	40		56	60	
Weight status (%)			0.674			0.196
Normal	16	16		14	14	
Overweight	46	40		50	39	
Obese	38	44		36	47	
**Paternal Characteristics**						
Age	47.72 ± 5.85	48.48 ± 5.30	0.300	48.41 ± 5.79	49.25 ± 6.32	0.245
Education (%)			0.923			0.412
Primary	10	9		13	12	
Secondary	63	65		63	72	
Higher	27	26		24	16	
Job status (%)			0.806			0.074
Employed	89	90		91	82	
Unemployed	11	10		9	18	
Smoking (%)			0.631			0.441
Yes	26	24		32	27	
No	74	76		68	73	
Physical activity (%)			0.249			0.661
Low	23	21		43	37	
Moderate	27	19		16	18	
High	50	60		41	45	
Weight status (%)			0.667			0.056
Normal	30	25		23	28	
Overweight	47	49		55	38	
Obese	23	26		22	34	

LST, Low screen time; HST, High screen time. Data are presented as mean ± SD for continuous variables and percentage for categorical variables.

Table 2 displays the socio-demographic, behavioral characteristics, and HRQoL in young men and women according to different screen time statuses during childhood. At adulthood (last re-examination follow-up), the mean ± SD values of age, BMI, physical activity, physical and mental HRQoL were 22.1 ± 2.2, 24.6 ± 4.7, 52.1 ± 5.7 and 47.9 ± 10.6, respectively. Except for age in women, there were no significant differences in education, marital status, occupation, smoking, and LTPA between men and women in the two groups of HST and LST. In general, the HRQoL scores in male participants were higher than in females in both mental and physical domains. Further sex-specific comparisons between HST and LST groups revealed poorer mental health (*P* value = 0.005), social functioning (*P* value = 0.013), and MCS (*P* value = 0.006) in males with higher screen time during their adolescence. Similar results were not observed in physical subscales in males and either physical or mental HRQoL in females.

**Table 2:** Adulthood Characteristics and Health-related Quality of Life According to Participants’ Sex and Childhood Screen Time: Tehran Lipid and Glucose Study, 2014-2018

	**Men**			**Women**		
	**LST**	**HST**	***P*** ** value**	**LST**	**HST**	***P*** ** value**
Age (y)	21.43 ± 2.26	21.70 ± 2.18	0.329	21.30 ± 2.15	22.09 ± 2.15	0.003
Education (%)			0.154			0.089
Diploma and less	75	68		65	55	
> Diploma	25	32		35	45	
Marital status (%)			0.383			0.880
Married	6	3		27	26	
Unmarried	94	97		73	74	
Occupation (%)			0.470			0.315
Employed	41	37		14	19	
Unemployed	59	63		86	81	
Smoking			0.100			0.265
Yes	23	32		5	8	
No	77	68		95	92	
LTPA (Met/h/wk)	18.97 ± 19.52	20.93 ± 26.27	0.483	12.43 ± 14.40	13.88 ± 17.45	0.519
BMI (kg/m^2^)	25.26 ± 4.52	25.08 ± 4.31	0.748	23.81 ± 4.45	24.47 ± 5.45	0.263
MH	78.17 ± 19.07	70.96 ± 24.09	0.005	71.99 ± 20.52	68.12 ± 21.08	0.168
RE	77.47 ± 21.02	72.35 ± 23.55	0.055	69.05 ± 21.53	68.80 ± 25.66	0.935
SF	87.65 ± 20.25	80.99 ± 25.58	0.013	82.78 ± 22.75	80.62 ± 26.04	0.492
VT	76.47 ± 20.80	72.31 ± 21.02	0.096	65.96 ± 23.21	60.24 ± 24.12	0.074
MCS	50.83 ± 9.41	47.47 ± 11.30	0.006	46.72 ± 10.14	45.18 ± 11.68	0.295
PF	96.85 ± 21.81	94.60 ± 14.02	0.185	94.79 ± 13.77	94.79 ± 15.14	0.252
GH	64.08 ± 23.47	63.67 ± 21.07	0.883	63.10 ± 21.49	58.19 ± 22.82	0.084
BP	86.57 ± 19.50	82.97 ± 21.54	0.145	80.81 ± 20.25	82.65 ± 19.71	0.485
RP	88.25 ± 17.87	85.15 ± 18.03	0.154	85.82 ± 16.39	83.93 ± 16.43	0.374
PCS	53.17 ± 5.86	53.12 ± 6.26	0.946	52.99 ± 5.34	52.54 ± 5.59	0.521

LST, Low screen time; HST, High screen time; MH, Mental health; RE, Role emotional; SF, Social functioning; VT, Vitality; MCS, Mental component summary; PF, Physical functioning; GH, General health; BP, Bodily pain; RP, Role physical; PCS, Physical component summary. Data are presented as mean ± SD for continuous variables and percentage for categorical variables.

Table 3 represents the adjusted association of HRQoL in young men and women with different childhood screen time. The current results showed that for men, HST in childhood was associated with decreased mental health (β = -6.41, 95% CI: -11.52, -1.3 and *P* = 0.014), social functioning (β = -5.9, 95% CI: -11.23, -0.57 and *P* = 0.03) and MCS (β = -2.86, 95% CI: -5.26, -0.45 and *P* = 0.02) even after adjusting for age, education, occupation, marital status, smoking, LTPA, and BMI.

**Table 3:** Association Between Health-related Quality of Life and Childhood Screen Time in Adult Men and Women: Adjusted Linear Regression Analysis

	**Men **		**Women **	
	**β (95% CI)**	***P*** ** Value**	**β (95% CI)**	***P*** ** Value**
MH	-6.41 (-11.52, -1.3)	0.014	-2.8 (-8.52, 2.93)	0.334
RE	-4.33 (-9.54, 0.89)	0.103	0.3 (-5.7, 6.3)	0.922
SF	-5.9 (-11.23, -0.57)	0.030	-1.35 (-7.69, 4.99)	0.674
VT	-2.93 (-7.84, 1.99)	0.242	-4.06 (-10.41, 2.29)	0.207
MCS	-2.86 (-5.26, -0.45)	0.020	-0.95 (-3.91, 2.01)	0.525
PF	-1.88 (-5.18, 1.42)	0.262	-1.86 (-5.37, 1.65)	0.297
GH	-0.16 (-5.46, 5.15)	0.954	-3.47 (-9.06, 2.11)	0.221
BP	-3.56 (-8.57, 1.45)	0.163	1.68 (-3.65, 7.01)	0.534
RP	-2.88 (-7.23, 1.48)	0.195	-1.86 (-6.14, 2.43)	0.394
PCS	-0.08 (-1.54, 1.38)	0.917	-0.43 (-1.84, 0.97)	0.542

LST, Low screen time; HST, High screen time; CI, confidence interval; MH, Mental health; RE, Role emotional; SF, Social functioning; VT, Vitality; MCS, Mental component summary; PF, Physical functioning; GH, General health; BP, Bodily pain; RP, Role physical; PCS, Physical component summary. β represents the difference in the means of the health-related quality of life score for the HST group compared to the LST group. The model was adjusted for participants’ age, education, occupation, marital status, smoking, leisure time physical activity, and BMI.

Finally, Table 4 shows the ORs of poor HRQoL in young men and women with HST compared to their counterparts with LST during adolescence. In men, the unadjusted OR of poor mental HRQoL was 1.99 (95% CI: 1.12- 3.56; *P* = 0.019) for the HST group compared to the LST group. After adjusting for all potential confounders, including age, education, occupation, marital status, smoking, LTPA, and BMI, the odds of poor MCS were significantly higher in those with HST compared to their counterparts with LST (OR = 1.95, 95% CI: 1.08-3.53; *P* = 0.027).

**Table 4:** Odds Ratios of Poor Quality of Life in Adult Men and Women Considering their Childhood Screen Time: Logistic Regression Analysis

	**MCS**		**PCS**	
	**OR (95% CI)**	***P*** ** value**	**OR (95% CI)**	***P*** ** value**
Boys				
Model 1	1.99 (1.12,3.56)	0.019	0.96 (0.54,1.69)	0.889
Model 2	1.99 (1.11,3.56)	0.020	0.93 (0.53,1.65)	0.808
Model 3	1.95 (1.08,3.53)	0.027	1.07 (0.58,1.96)	0.832
Girls				
Model 1	1.33 (0.71,2.49)	0.369	1.55 (0.83,2.92)	0.169
Model 2	1.24 (0.65,2.36)	0.515	1.59 (0.84,3.04)	0.156
Model 3	1.17 (0.61,2.25)	0.643	1.73 (0.89,3.37)	0.105

MCS, Mental component summary; PCS, Physical component summary. OR represents the odds of poor quality of life for high screen time compared to low screen time. CI represents the confidence interval. Model 1: unadjusted. Model 2: age-adjusted. Model 3: adjusted for adulthood age, education, occupation, marital status, smoking, leisure time physical activity, and BMI.

## Discussion

 The present study is the first attempt to investigate the association between adolescents’ screen time and their HRQoL in young adulthood. After adjusting for potential confounders, the current results confirmed that compared to those who were exposed to lower screen time across adolescence, boys with higher screen time reported lower MCS as adults. Our results showed that spending more hours on screen during adolescence led boys to experiencing poorer social functioning and mental health in adulthood. However, girls’ higher screen time was not significantly related to their adulthood MCS. Unexpectedly, we observed no significant relationship between adolescents’ higher screen time and their PCS in adulthood in either sex.

The present results revealed that higher screen time during adolescence could negatively impact the males’ mental HRQoL in adulthood. In the digital era, it is not a secret that many people are exposed to screens, including electronic devices, electronic games, and television. Adolescents are no exception, and screen time has also increased dramatically among the young generations.^3^ The problem is that spending much time in front of the screen could decrease one’s psychological well-being.^29-31^ A survey conducted in the United States showed that more hours of daily screen time during adolescence were related to less curiosity, lower self-control, more distractibility, more difficulty making friends, less emotional stability, anxiety, and depression.^9^ Another study found that HST was associated with poorer mental health outcomes in youth.^32^ Two cross-sectional studies from Australia and Iran observed that more screen time was associated with lower scores in the adolescents’ mental and physical components of HRQoL.^14,15^ Based on the current findings, adolescent boys exposed to higher screen time reported poorer MCS as well as lower scores in two of MCS’s subscales, including mental health and social functioning in adulthood. Gender differences were also observed in a previous study examining the impact of physical activity and screen time on adults’ HRQoL. This study showed the stronger influence of screen time on males’ HRQoL.^33^ However, our results only revealed a gender-based difference when we assessed the association between screen time and the mental components of HRQoL. A possible hypothesis in the interpretation of this result can be the different nature of screen viewing in Iranian boys and girls at the time of its measurement in the present study: in those years, the higher prevalence of computer games in teenage boys compared to their female counterparts may have had a different impact on their evaluation of health. Also, the gender difference observed in this study and its difference with the results of other studies may be accounted for during the follow-up period of the current study. While the aforementioned study examined the cross-sectional influence of screen time in adults, our study explored the impact of adolescents’ screen time on their adulthood HRQoL. Overall, since none of the previous studies investigated the association between screen time across adolescence and HRQoL in adulthood, comparing the current results with prior findings seems difficult. Nevertheless, these hypotheses require further investigation and documentary evidence.

In the present study, in order to determine the participants’ screen time, they were asked how many hours a day they spent watching television and playing video (or computer) games. As a study indicates, the association between different types of screen time and mental health problems is varied.^31^ It seems that not all types of screen-based activities have the same impact on mental health. According to previous studies, excessive playing of video games is among screen-based activities which can adversely affect psychosocial outcomes.^34,35^ It has been shown that excessive playing of video games was related to lower psychosocial well-being, loneliness,^36^ lack of real-life friends,^37^ stress, and maladaptive coping.^38^ The striking point is that, as the literature shows, males start playing video games at younger ages, play more frequently, and spend more time gaming than females.^39^ Thus, we hypothesized that a possible explanation for our findings might be related to the fact that boys usually spend more time in front of the screen playing video games,^40,41^ which might negatively impact their psychosocial well-being. For example, a study conducted in China revealed that too much video gaming caused boys to experience worse mental health outcomes.^29^ Furthermore, evidence shows that those who spend more time on video gaming have more problems with peers and lower social skills.^42^ Therefore, our second hypothesis is that limited social skills might be a potential reason for the poorer social functioning of the present study’s male participants. Unfortunately, we were unable to quantify the time our participants spent watching television and playing video games separately, so we could not investigate the effect of the type of screen-based activities on HRQoL.

Unlike our findings, which indicated no significant relationship between higher screen time during adolescence and PCS in adulthood, many other studies have reported a significant association between prolonged screen time and adverse physical health outcomes.^43-45^ For instance, a systematic review found that excessive screen time was linked with poor sleep and potential risks for cardiovascular diseases, obesity, impaired vision, and reduced bone density.^43^ Two potential reasons may justify the difference between our results and previous findings. First, most prior studies concentrated on screen time’s influence on children and adolescents’ health outcomes. Second, none tracked the impact of adolescents’ screen time on HRQoL in adulthood.

The main strength of our research is that, to our knowledge, for the first time, we investigated the influence of adolescents’ screen time on HRQoL in adulthood. Despite the high prevalence of screen time among adolescents, there is no clear understanding regarding the impact of too much screen time during adolescence on later health-related outcomes. Hence, our study may shed light on this area. However, we also encountered some limitations. Although we had general information on how many hours the participants spent watching television and playing video (or computer) games, these data were not separated. In other words, we lacked information regarding the exact time spent on either of the abovementioned screen-based activities. Since the type of screen-based activities might have different influences on mental and physical health,^43^ we suggest that future studies consider this issue. Moreover, our results cannot be generalized to the rural populations because the current data was collected from an urban sample.

## Conclusion

 In conclusion, our results showed that boys exposed to higher screen time during adolescence were more likely to report lower scores in the mental domains of HRQoL, including social functioning and mental health in adulthood. These results highlight the influence of adolescent boys’ excessive screen time on their adulthood mental HRQoL. The current results indicate the need for intervention programs for teenage boys to decrease their screen-based activities in order to prevent possible negative mental health outcomes in older ages. Finally, since this is the first study that examined the association between adolescents’ screen time and their HRQoL in young adulthood, further studies are needed to clarify this relationship.

## Supplementary Files


Supplementary file 1 contains Table S1.
