# Supplementary material for: Self-rated Health in Youth with Different Screen Time in Their Adolescence: Tehran Lipid and Glucose Study
Source: Arch Iran Med. 2023 Dec 1;26(12):671–8. doi: 10.34172/aim.2023.99 (PMC10915920; doi:10.34172/aim.2023.99)
Supplement: Supplementary file 1 — contains Table S1. [file aim-26-671-s001.pdf]

Arch Iran Med. December 2023;26(12):671-S1
10.34172/aim.2023.99

Supplementary file 1
Table S1. Percentage of Missing Data on Individual and Parental Characteristics

**Childhood Characteristics**	Percentage
Age	0 %
Education	0 %
LTPA (Met/h/week)	35 %
BMI	1 %
Screen time	9 %
**Early adulthood characteristics**	
Age	0 %
Education	0 %
Occupation	0 %
Marital status	0 %
Smoking	1.4 %
LTPA	38 %
BMI	0 %
**Maternal characteristics**	
Age	7.2 %
Education	7.5 %
Job status	7.3 %
Smoking	7.5 %
Physical activity	8 %
Weight status	8.4 %
**Paternal characteristics**	
Age	18.1 %
Education	18.1 %
Job status	18.2 %
Smoking	18.2 %
Physical activity	19.2 %
Weight status	18.7 %
